# Hybrid assembled genomes of three *Salmonella enterica* isolates from municipal wastewater in Tennessee, USA

**DOI:** 10.1128/mra.01271-24

**Published:** 2025-06-27

**Authors:** Jia Wang, Lauren K. Hudson, Daniel W. Bryan, Thomas G. Denes

**Affiliations:** 1 Department of Food Science, The University of Tennessee, Knoxville, Tennessee, USA; University of Maryland School of Medicine, Baltimore, Maryland, USA

**Keywords:** *Salmonella*, wastewater, surveillance

## Abstract

Three *Salmonella enterica* isolates were isolated from a municipal wastewater treatment plant in Knoxville, TN, USA. Genomic DNA was extracted and sequenced on the Illumina and Oxford Nanopore platforms. Annotated hybrid assemblies showed the strains were predicted to be serotypes Bareilly (*n* = 2) and Typhimurium (*n* = 1).

## ANNOUNCEMENT

*Salmonella enterica* is a globally widespread bacterial pathogen, causing an estimated 100 million infections and over 200,000 deaths annually (1, 2). Reporting novel *S. enterica* strains from municipal wastewater contributes genomic resources for comparative analyses and genomic surveillance. This study presents the complete and draft genome sequences of three *S. enterica* strains (UTK W1-0001, UTK W1-0003, and UTK W1-0011) isolated from municipal wastewater samples collected at a local wastewater treatment plant in Knoxville, Tennessee, USA.

Samples were collected from the primary effluent of municipal wastewater on 9 October 2023. The collected wastewater samples were filtered and enriched for *Salmonella* spp. following the modified Food and Drug Administration’s Bacteriological Analytical Manual protocol (3, 4). Single colonies were isolated on selective media, and pure cultures were prepared. The presumptive *Salmonella* isolates were cultured overnight in brain heart infusion broth at 37°C with shaking at 200 rpm. DNA was extracted by the DNeasy Blood and Tissue Kit (Qiagen), assessed with Nanodrop (Thermo Fisher Scientific) and Qubit 4 (Thermo Fisher Scientific), and shipped to SeqCenter (Pittsburgh, PA, USA). Illumina short-read sequencing was performed using the Illumina DNA Prep kit and NovaSeq X Plus sequencer (Illumina), producing 151 bp paired-end reads (5). For Nanopore sequencing, DNA was extracted using the Zymo Quick-DNA HMW MagBead kit (Zymo Research Corp.). The library was prepared using the Oxford Nanopore rapid barcoding kit (SQK-RBK114.24) and sequenced with a MinION sequencer with Flongle (Oxford Nanopore Technologies). Basecalling was performed by MinKNOW (v24.06.5) using the fast basecalling model with adapter trimming, default quality score threshold, and no size-selection (6). Read quality was checked by FastQC v0.11.9 (7), and trimming was performed by Trimmomatic v0.39 (8). The hybrid assembly was performed using Unicycler v0.4.8 in bold mode, including overlap identification and trimming, with rotation to start at dnaA/repA (9). For all software, default parameters were used except where otherwise noted.

BLAST analysis (10) of the 7 kb contig in UTK W1-0011 assembly revealed 96.7% nucleotide identity across 81% of the query sequence length to plasmid NZ_CP128883.1. kSNP4 (v4.1) was used to identify core genome single-nucleotide polymorphism (SNP) among these three *Salmonella* isolates (11), revealing each was a distinct strain. Genome sequencing data and annotation features are summarized in Table 1. The genomes of strains UTK W1-0001, UTK W1-0003, and UTK W1-0011 were predicted to have 151-152 virulence factor genes. Key virulence determinants included fimbrial and nonfimbrial adherence determinants, macrophage inducible gene mig-14, and type III secretion system (T3SS) encoded by *Salmonella* pathogenicity island 1 (SPI-1) and 2 (SPI-2). Compared with previously isolated *Salmonella enterica* strains harboring 100-130 virulence genes identified by VFDB (12–15), the three newly isolated *Salmonella enterica* strains exhibit a notable virulence profile potentially indicative of aggressive pathogenic characteristics.

**TABLE 1:** Genomic sequence statistics and features of three *S. enterica* isolates from a local wastewater treatment plant

Strain	No. of Illumina reads	No. of ONT reads	N50 of ONT reads	Total length (bp)^*a*^	No. of chromosome contigs^*a*^	No. of plasmid contigs^*a*^	GC content (%)^*a*^	Predicted serotype^*b*^	Illumina read coverage (×)^*c*^	Nanopore read coverage (×)^*c*^	No. of CDSs^*d*^	No. of tRNAs^*d*^	No. of CRISPR arrays^*e*^	Antibiotic resistance genes^*f*^
UTK W1-0001	2,419,110	5,000	19,045	4,964,787	1	1	52.17	Typhimurium	66	9	4,739	86	4	*aac(6')-Iaa, aph (6)-Id, blaCMY-2, sul2, tet(A), floR*
UTK W1-0003	3,066,142	8,000	17,340	4,843,513	1	1	52.05	Bareilly	90	14	4,648	85	3	*aac(6')-Iaa*
UTK W1-0011	2,943,720	7,000	17,533	5,032,162	1	2	52.14	Bareilly	83	12	4,871	86	2	*aac(6')-Iaa*

^*a*^ Assembly statistics and quality were generated using QUAST v5.2.0 (16) and CheckM v1.1.3 (17).

^*b*^ The species and serotype were analyzed by EnteroBase v1.2.0 (18).

^*c*^ Average read coverage was determined using Minimap2 v2.14 (19), BBMap v38.63 (20), and SAMtools v1.15.1 (21).

^*d*^ The assemblies were annotated by the NCBI Prokaryotic Genome Annotation Pipeline (PGAP, v6.8) (22).

^*e*^ The clustered regularly interspaced short palindromic repeat (CRISPR) arrays were predicted using CRISPRFinder (23, 24).

^*f*^ Antimicrobial resistance and virulence genes were screened using ResFinder v4.6.0 (25) and Virulence Factor Database (VFDB) (26).

## Data Availability

The genomic sequencing data and assemblies for strains UTK W1-0001, UTK W1-0003 and UTK W1-0011 have been deposited in the NCBI database under BioProject number PRJNA1169800. The corresponding BioSample numbers are SAMN44082895, SAMN44082896, and SAMN44082897; SRA accession numbers are SRR30900535, SRR30900538, SRR30900534, SRR30900537, SRR30900533, and SRR30900536. Assembly accession numbers are CP171353.1, CP171354.1, CP171351.1, CP171352.1, and JBJDRN000000000.1.

## References

[1] Wang BX, Butler DSC, Hamblin M, Monack DM. 2023. One species, different diseases: the unique molecular mechanisms that underlie the pathogenesis of typhoidal Salmonella infections. Curr Opin Microbiol 72:102262. doi:10.1016/j.mib.2022.102262 36640585 PMC10023398

[2] Sears KT, Galen JE, Tennant SM. 2021. Advances in the development of Salmonella-based vaccine strategies for protection against Salmonellosis in humans. J Appl Microbiol 131:2640–2658. doi:10.1111/jam.15055 33665941 PMC9292744

[3] Strawn LK, Gröhn YT, Warchocki S, Worobo RW, Bihn EA, Wiedmann M. 2013. Risk factors associated with Salmonella and Listeria monocytogenes contamination of produce fields. Appl Environ Microbiol 79:7618–7627. doi:10.1128/AEM.02831-13 24077713 PMC3837806

[4] Strawn LK, Fortes ED, Bihn EA, Nightingale KK, Gröhn YT, Worobo RW, Wiedmann M, Bergholz PW. 2013. Landscape and meteorological factors affecting prevalence of three food-borne pathogens in fruit and vegetable farms. Appl Environ Microbiol 79:588–600. doi:10.1128/AEM.02491-12 23144137 PMC3553790

[5] Wang J, Schamp CN, Hudson LK, Chaggar HK, Bryan DW, Garman KN, Radosevich MA, Denes TG. 2025. Whole-genome sequencing and metagenomics reveals diversity and prevalence of soil Listeria spp. in the Nantahala National Forest. Microbiol Spectr 13:e0171224. doi:10.1128/spectrum.01712-24 39651889 PMC11705966

[6] Technologies ON. 2025. MinKNOW. Available from: https://nanoporetech.com/document/experiment-companion-minknow

[7] Andrews S. 2010. FastQC: a quality control tool for high throughput sequence data, on Babraham Bioinformatics, Babraham Institute, Cambridge, United Kingdom. Available from: https://www.bioinformatics.babraham.ac.uk/projects/fastqc

[8] Bolger AM, Lohse M, Usadel B. 2014. Trimmomatic: a flexible trimmer for Illumina sequence data. Bioinformatics 30:2114–2120. doi:10.1093/bioinformatics/btu170 24695404 PMC4103590

[9] Wick RR, Judd LM, Gorrie CL, Holt KE. 2017. Unicycler: resolving bacterial genome assemblies from short and long sequencing reads. PLOS Comput Biol 13:e1005595. doi:10.1371/journal.pcbi.1005595 28594827 PMC5481147

[10] Altschul SF, Gish W, Miller W, Myers EW, Lipman DJ. 1990. Basic local alignment search tool. J Mol Biol 215:403–410. doi:10.1016/S0022-2836(05)80360-2 2231712

[11] Hall BG, Nisbet J. 2023. Building phylogenetic trees from genome sequences with kSNP4. Mol Biol Evol 40:msad235. doi:10.1093/molbev/msad235 37948764 PMC10640685

[12] Tiengo A, Orsini M, Petrin S, Losasso C, Longo A, Cento G, Ciot L, Ceruti R, Cibin V, Barco L. 2023. Whole-genome sequence of Salmonella enterica serovar Bispebjerg from Turkey reveals its pathogenic potential. Microbiol Resour Announc 12:e0004323. doi:10.1128/mra.00043-23 37022181 PMC10190257

[13] Popy NN, Hoque MN, Khan MFR, Biswas L, Rahman MH, Saiduzzaman M, Rahman M, Rahman MB. 2024. Draft genome sequencing of a multidrug-resistant Salmonella enterica subspecies enterica serovar Typhimurium strain isolated from chicken in Bangladesh. Microbiol Resour Announc 13:e0061923. doi:10.1128/mra.00619-23 38088574 PMC10793348

[14] Schlund O, Göhler A, Borowiak M, Deneke C, Fischer M, Lamparter MC. 2022. Complete genome sequence of a Salmonella enterica subsp. enterica serovar Tennessee strain from Tahini. Microbiol Resour Announc 11:e0040722. doi:10.1128/mra.00407-22 35894624 PMC9387254

[15] Helmy Yosra A, Kabir A, Saleh M, Kennedy Laura A, Burns L, Johnson B. 2024. Draft genome sequence analysis of multidrug-resistant Salmonella enterica subsp. enterica serovar Mbandaka harboring colistin resistance gene mcr-9.1 isolated from foals in Kentucky, USA. Microbiol Resour Announc 13:e00737–24. doi:10.1128/mra.00737-24 39373480 PMC11556123

[16] Gurevich A, Saveliev V, Vyahhi N, Tesler G. 2013. QUAST: quality assessment tool for genome assemblies. Bioinformatics 29:1072–1075. doi:10.1093/bioinformatics/btt086 23422339 PMC3624806

[17] Parks DH, Imelfort M, Skennerton CT, Hugenholtz P, Tyson GW. 2015. CheckM: assessing the quality of microbial genomes recovered from isolates, single cells, and metagenomes. Genome Res 25:1043–1055. doi:10.1101/gr.186072.114 25977477 PMC4484387

[18] Zhou Z, Alikhan N-F, Mohamed K, Fan Y, Achtman M, Brown D, Chattaway M, Dallman T, Delahay R, Kornschober C. 2020. The EnteroBase user's guide, with case studies on Salmonella transmissions, Yersinia pestis phylogeny, and Escherichia core genomic diversity. Genome Res 30:138–152. 31809257 10.1101/gr.251678.119 PMC6961584

[19] Li H. 2018. Minimap2: pairwise alignment for nucleotide sequences. Bioinformatics 34:3094–3100. doi:10.1093/bioinformatics/bty191 29750242 PMC6137996

[20] Bushnell B. 2015. BBMap short-read aligner, and other bioinformatics tools. University of California, Berkeley, CA.

[21] Li H, Handsaker B, Wysoker A, Fennell T, Ruan J, Homer N, Marth G, Abecasis G, Durbin R, Genome Project Data Processing S. 2009. The sequence alignment/map format and SAMtools. Bioinformatics 25:2078–2079. doi:10.1093/bioinformatics/btp352 19505943 PMC2723002

[22] Tatusova T, DiCuccio M, Badretdin A, Chetvernin V, Nawrocki EP, Zaslavsky L, Lomsadze A, Pruitt KD, Borodovsky M, Ostell J. 2016. NCBI prokaryotic genome annotation pipeline. Nucleic Acids Res 44:6614–6624. doi:10.1093/nar/gkw569 27342282 PMC5001611

[23] Grissa I, Vergnaud G, Pourcel C. 2007. CRISPRFinder: a web tool to identify clustered regularly interspaced short palindromic repeats. Nucleic Acids Res 35:W52–W57. doi:10.1093/nar/gkm360 17537822 PMC1933234

[24] Couvin D, Bernheim A, Toffano-Nioche C, Touchon M, Michalik J, Néron B, Rocha EPC, Vergnaud G, Gautheret D, Pourcel C. 2018. CRISPRCasFinder, an update of CRISRFinder, includes a portable version, enhanced performance and integrates search for Cas proteins. Nucleic Acids Res 46:W246–W251. doi:10.1093/nar/gky425 29790974 PMC6030898

[25] Zankari E, Hasman H, Cosentino S, Vestergaard M, Rasmussen S, Lund O, Aarestrup FM, Larsen MV. 2012. Identification of acquired antimicrobial resistance genes. J Antimicrob Chemother 67:2640–2644. doi:10.1093/jac/dks261 22782487 PMC3468078

[26] Chen L, Zheng D, Liu B, Yang J, Jin Q. 2016. VFDB 2016: hierarchical and refined dataset for big data analysis--10 years on. Nucleic Acids Res 44:D694–D697. doi:10.1093/nar/gkv1239 26578559 PMC4702877

